# HTLV-1 Tax Specific CD8+ T Cells Express Low Levels of Tim-3 in HTLV-1 Infection: Implications for Progression to Neurological Complications

**DOI:** 10.1371/journal.pntd.0001030

**Published:** 2011-04-26

**Authors:** Lishomwa C. Ndhlovu, Fabio E. Leal, Aaron M. Hasenkrug, Aashish R. Jha, Karina I. Carvalho, Ijeoma G. Eccles-James, Fernanda R. Bruno, Raphaella G. S. Vieira, Vanessa A. York, Glen M. Chew, R. Brad Jones, Yuetsu Tanaka, Walter K. Neto, Sabri S. Sanabani, Mario A. Ostrowski, Aluisio C. Segurado, Douglas F. Nixon, Esper G. Kallas

**Affiliations:** 1 Division of Experimental Medicine, Department of Medicine, University of California San Francisco, San Francisco, California, United States of America; 2 Division of Clinical Immunology and Allergy, Department of Infectious Diseases, School of Medicine, University of São Paulo, São Paulo, Brazil; 3 Department of Infectious Diseases, School of Medicine, University of São Paulo, São Paulo, Brazil; 4 Department of Immunology, University of Toronto, Toronto, Ontario, Canada; 5 Department of Immunology, University of the Ryukyus, Okinawa, Japan; 6 Molecular Biology Laboratory, Fundação Pró-Sangue, Hemocentro de São Paulo, São Paulo, Brazil; Centre for Cellular and Molecular Biology (CCMB), India

## Abstract

The T cell immunoglobulin mucin 3 (Tim-3) receptor is highly expressed on HIV-1-specific T cells, rendering them partially “exhausted” and unable to contribute to the effective immune mediated control of viral replication. To elucidate novel mechanisms contributing to the HTLV-1 neurological complex and its classic neurological presentation called HAM/TSP (HTLV-1 associated myelopathy/tropical spastic paraparesis), we investigated the expression of the Tim-3 receptor on CD8^+^ T cells from a cohort of HTLV-1 seropositive asymptomatic and symptomatic patients. Patients diagnosed with HAM/TSP down-regulated Tim-3 expression on both CD8^+^ and CD4^+^ T cells compared to asymptomatic patients and HTLV-1 seronegative controls. HTLV-1 Tax-specific, HLA-A*02 restricted CD8^+^ T cells among HAM/TSP individuals expressed markedly lower levels of Tim-3. We observed Tax expressing cells in both Tim-3^+^ and Tim-3^−^ fractions. Taken together, these data indicate that there is a systematic downregulation of Tim-3 levels on T cells in HTLV-1 infection, sustaining a profoundly highly active population of potentially pathogenic T cells that may allow for the development of HTLV-1 complications.

## Author Summary

The retrovirus, Human T lymphotropic virus type 1 (HTLV-1) infects 10–20 million people worldwide. The majority of infected individuals are asymptomatic; however, approximately 3% develop the debilitating neurological disease, HTLV-1-associated myelopathy/tropical spastic paraparesis (HAM/TSP). There is also currently no cure, vaccine or effective therapy for HTLV-1 infection. The precise role of CD8+ killer T cells in the control or contribution of HTLV-1 disease progression remains unclear. The T-cell immunoglobulin mucin domain-containing (Tim) proteins are type 1 transmembrane proteins. Three human Tim proteins (Tim-1, -3, and -4) exist and display markedly diverse expression patterns and functions. Tim-3 is upregulated on CD8^+^ T cells during chronic viral infections leading to a population of poorly functioning T cells. We investigated the expression of Tim-3 on T cells from patients with asymptomatic and symptomatic HTLV-1 infection and compared this with HTLV-1 uninfected donors. Patients diagnosed with HAM/TSP down-regulated Tim-3 expression on T cells when compared to asymptomatic patients and uninfected controls. Our study provides evidence of a novel mechanism for the persistent inflammation observed in HTLV-1 infected patients with neurological deficits and significantly advances our understanding of how the Tim-3 pathway functions.

## Introduction

The vast majority of HTLV-1-infected individuals with low and stable HTLV-1 proviral load levels are clinically asymptomatic for life [1]. However, 1–3% of subjects develop progressive neurological complications related to HTLV-1 infection, classically denominated as HTLV-1 associated myelopathy/tropical spastic paraparesis (HAM/TSP) [2], [3], [4]. The infection can also lead to a debilitating malignancy, known as HTLV-1 associated adult T cell leukemia (ATL) in approximately 2–5% of infected individuals [4], [5], [6], [7].

The immune response, and in particular the cellular immune response, plays an important role in the control of HTLV-1 infection [8], [9], [10], [11], [12]. *In vitro* studies further demonstrate that CD8^+^ T cell responses are able to directly lyse HTLV-1-infected CD4^+^ T cells [9], [11], [13]. In patients with HAM/TSP, CD8^+^ T cells are capable of producing multi-cytokine responses and are able to release cytotoxic molecules [14], [15]. Recent studies have selected out patients with HLA-A*02 and HLA-Cw08 genes as being associated with lower HTLV-1 proviral load and a reduced risk of progression to HAM/TSP [16], [17].

While these data support an important protective role for the CD8^+^ T cell immune response with the potential for viral control, other studies suggest that HTLV-1-specific CD8^+^ T cells may paradoxically contribute to the neuromuscular immunopathology through autoimmune mechanisms, leading to the clinical manifestation of HAM/TSP [18]. Furthermore, patients with HAM/TSP also present with high numbers of HTLV-1 Tax-specific CD8+ T cells in the cerebrospinal fluid [15], [19], [20], [21], [22] that are thought to play a immunopathogenic role, either by release of neurotoxic cytokines, such as TNF-α and IFN-γ [23], [24], or by direct cytotoxicity. It is evident from these studies that the precise role of CD8^+^ T cells in the control or pathogenesis of HTLV-1 disease progression remain unclear. Further knowledge of the mechanisms leading to T cell induced immunopathology in HTLV-1 infection will be important in determining successful immune-based therapies and provide insights for effective vaccine designs.

During chronic viral infections, virus-specific CD8^+^ T cells undergo an altered pattern of differentiation and can become exhausted [25], [26]. CD8^+^ T cell exhaustion is a transcriptionally altered state of T cell differentiation distinct from functional effector or memory CD8^+^ T cells [27]. CD8^+^ T cell exhaustion leads to profound T cell dysfunction and the inability of the T cells to control retroviral replication [28], [29], [30]. Conversely, downregulation of exhaustion markers could lead to a highly functional population of T cells. T cell immunoglobulin and mucin domain-containing protein 3 (Tim-3), is upregulated on CD8^+^ T cells during chronic viral infections [29], [30], [31], [32], [33], [34], [35], [36]. Programmed death receptor-1 (PD-1) is also known as another immune exhaustion biomarker expressed in chronic viral infections [28], [37], [38], [39], [40], [41], [42], [43]. High levels of PD-1 and Tim-3 on virus-specific T cells have been shown to lead to poor proliferative capacity and, in some cases, ineffective Th1 cytokine production [29], [39], [44]. A sustained downregulation of these receptors would lead to an exacerbated constitutively active T cell population. The phenotypic profile of immune exhaustion markers on T cells is unknown in seropositive HTLV-1 individuals. In this study, we show for the first time that HTLV-1 associated complications may be related to the highly responsive inflammatory Tax-specific T cells in HTLV-1-infected individuals. These results support the idea that HTLV-1 infection induces mechanisms resulting in a limited T cell exhaustion profile, leading potentially to neuro-immunopathology and disease complications.

## Materials and Methods

### Ethics Statement

The research involving human participants reported in this study was approved by the institutional review board of the University of Sao Paulo (IRB #0855/08) Sao Paulo, Brazil. Informed consent was obtained for all subjects. All clinical investigation were conducted according to the principles expressed in the Declaration of Helsinki (http://www.wma.net/en/30publications/10policies/b3/index.html).

### Humans Subjects

Patients were serially recruited in the HTLV-1 Outpatient Clinic at the University of Sao Paulo, Brazil in two stages with written informed consent approved by the University of Sao Paulo's Institutional Review Board (#0855/08). The diagnosis of HAM/TSP based on criteria outlined by the WHO [45] (Table 1). The majority of the patients were female (63%) with a median age of 48 (IQR: 22–66) years. We enrolled age and sex matched healthy uninfected volunteers without clinical and laboratory evidence of HTLV-1-associated disease, from the same demographics as the infected subjects. All HTLV -1 seropositive subjects tested negative for Hepatitis B, Hepatitis C, and HIV infections. No other inflammatory diseases or disorders were present in any of the participants. Blood samples were processed with Ficoll-Paque PLUS (Amersham Pharmacia Biotech, Uppsala, Sweden) gradient centrifugation, and peripheral-blood mononuclear cells (PBMC) were isolated and cyropreserved in fetal bovine serum (FBS) containing 10% DMSO in liquid nitrogen.

**Table 1:** Patients description.

ID Number	Gender	Age	Clinical Presentation	PBMC	HLA-A*02
		(years)		(cps/1000)	Status
237	M	39	asymptomatic	20	pos
410	F	43	asymptomatic	14	pos
411	F	47	asymptomatic	84	pos
405	F	22	asymptomatic	15	pos
403	F	53	asymptomatic	604	pos
240	F	N/A	asymptomatic	0	pos
425	M	29	asymptomatic	43	
416	M	48	asymptomatic	140	pos
221	M	N/A	asymptomatic	9	pos
424	M	46	asymptomatic	106	
418	M	66	asymptomatic	<1	
419	F	33	asymptomatic	72	
421	M	54	asymptomatic	23	
423	F	42	asymptomatic	72	
218	F	46	HAM/TSP	2	pos
402	F	50	HAM/TSP	152	pos
224	F	57	HAM/TSP	1923	pos
412	F	53	HAM/TSP	117	pos
312	F	N/A	HAM/TSP	161	pos
413	F	61	HAM/TSP	1510	pos
420	M	64	HAM/TSP	12	
422	F	64	HAM/TSP	ND	
HD1	N/A	N/A	Healthy		
HD2	F	46	Healthy		
HD3	F	39	Healthy		
HD4	F	29	Healthy		
HD5	F	60	Healthy		
HD6	M	37	Healthy		
HD7	F	45	Healthy		

ND = not detected, N/A = not available.

### Pentamers, Peptides and Cytokines

Conjugated Pentamers were obtained commercially from Proimmune (Oxford, UK). The HLA-A*02 restricted HTLV-1 Tax (LLFGYPVYV) and CMV (NLVPMVATV) peptides were obtained from New England Peptide (Gardner, MA). In some experiments rIL-2 [80 IU/ml] (Roche Diagnostics, Mannheim, Germany) and rIL-15 [50 ng/ml] (R&D Systems, Minneapolis, MN) were used during *in vitro* culture studies.

### Flow Cytometry Assessment

Cryopreserved PBMC were rapidly thawed in warm RPMI 1640 with 10% FBS, washed in FACS buffer (PBS, with 0.5% bovine serum albumin, 2 mM EDTA (Sigma-Aldrich, St. Louis, MO)). For staining, 5×10^5^ cells were incubated with conjugated antibodies against Tim-3 (R&D Systems, Minneapolis, MN), PD-1 (Biolegend, San Diego, CA), CD4, CD8, CD3 (all from BD Biosciences, San Jose, CA) for 30 min on ice. In some experiments, PMBC were then fixed and permeabilized prior to staining with conjugated anti-Tax (clone Lt-4) antibodies [46] or a control labeled IgG. Fluorescence minus one (FMO) samples were prepared for each fluorochrome to facilitate gating as well as conjugated isotype control antibodies. Anti-mouse IgG-coated beads were stained with each fluorochrome separately and used for software-based compensation. Analysis was performed using a FACSCanto instrument (BD Biosciences) and at least 100,000 events were collected and analyzed with FlowJo software (TreeStar, Ashland, OR).

To define pentamer positive cells: staining was initially performed immediately after thawing with biotin-labeled HLA-A2 Tax or CMV epitope specific pentamer fluorotags followed a secondary staining step with fluorophore conjugated antibodies against CD8 (BD), Tim-3 (R&D Systems), PD-1 (Biolegend) and CD3 (BD), and with labeled streptavidin. Cells were washed twice with PBS containing 1% FBS, then fixed in 2% paraformaldehyde and run on a customized BD FACSCanto within 12 hours.

### Viral Load Assessment

HTLV-1 proviral DNA was extracted from PBMC using a commercial kit (Qiagen GmbH, Hilden Germany) and according to the manufacturer's instructions. The extracted DNA was used as a template to amplify a fragment of 158 bp from the viral tax region using previously published primers[47]. The SYBR green real-time PCR assay was carried out in 25 µl PCR mixture containing 10× Tris (pH 8.3; Invitrogen, Brazil), 1.5 mM MgCl_2_, 0.2 µM of each primer, 0.2 mM of each dNTPs, SYBR Green (18.75 Units/r×n; Cambrex Bio Science, Rockland, ME) and 1 unit of platinum Taq polymerase (Invitrogen, Brazil). The amplification was performed in the Bio-Rad iCycler iQ system using an initial denaturation step at 95°C for 2 minutes, followed by 50 cycles of 95°C for 30 seconds, 57°C for 30 seconds and 72°C for 30 seconds. The human housekeeping β globin gene primers GH20 and PC04[48] were used as an internal control calibrator. For each run, standard curves for the value of HTLV-1 tax were generated from MT-2 cells of log_10_ dilutions (from 10^5^ to 10^0^ copies). The threshold cycle for each clinical sample was calculated by defining the point at which the fluorescence exceeded a threshold limit. Each sample was assayed in duplicate and the mean of the two values was considered as the copy number of the sample. The amount of HTLV-1 proviral load was calculated as follows: copy number of HTLV-1 (tax) per 1,000 cells  =  (copy number of HTLV-1 tax)/(copy number of β globin/2) ×1,000 cells. The method could detect 1 copy per 10^3^ PBMC.

### Elispot Assays

MAIPS4510 Elispot plates (Millipore, Danvers, MA) were coated with anti-IFN-γ (10 µg/ml) (Mabtech, Nacka Strand, Sweden) in PBS, 50 µl/well, either overnight at 4°C or for one hour at room temperature. After three washes with PBS, PBMC (1×10^5^ cells/well) and the appropriate antigens were added (Tax peptide and CMV peptide), with a final volume of 200 µl/well. Plates were incubated at 37°C in 5% CO_2_ for 16–20 hours. After washing with phosphate-buffered saline (PBS) plus 0.1% Tween 20 (PBST), biotinylated anti-IFN-γ 1 µg/ml) (Mabtech), antibodies were added to the appropriate wells in PBS 0.1% tween 1% BSA (PBSTB) for 30 minutes at room temperature. Plates were washed again three times with PBST, and alkaline phosphatase-conjugated streptavidin (Jackson Immunoresearch, West Grove, PA) was added (50 µl of 1∶1,000 dilution in PBSTB) and incubated for 30 min at room temperature. Plates were washed in PBSTB, soaked for 1 hour in PBSTB and incubated with blue substrate (Vector Labs, Burlingame, CA) until spots were clearly visible, then rinsed with tap water. When plates were dry, spots were counted using an automated ELISPOT reader.

### Statistical Analysis

Statistical analysis was performed by using GraphPad Prism statistical software (GraphPad Software, San Diego, CA). Non-parametric statistical tests were used. The Mann-Whitney U was used for comparison tests and the Spearman rank test were used for correlation analyses.

## Results

### Subjects

Peripheral venous blood was drawn from 22 HTLV-1 seropositive patients and 7 HTLV-1 seronegative matched donors, all screened for the presence of HLA-A*02 alleles, and peripheral blood mononuclear cells (PBMC) were extracted and cryopreserved.

### Tim-3 and PD-1 Expression on CD8+ and CD4+ T Cells in Patients with HTLV-1 Infection

**Figure 1:**
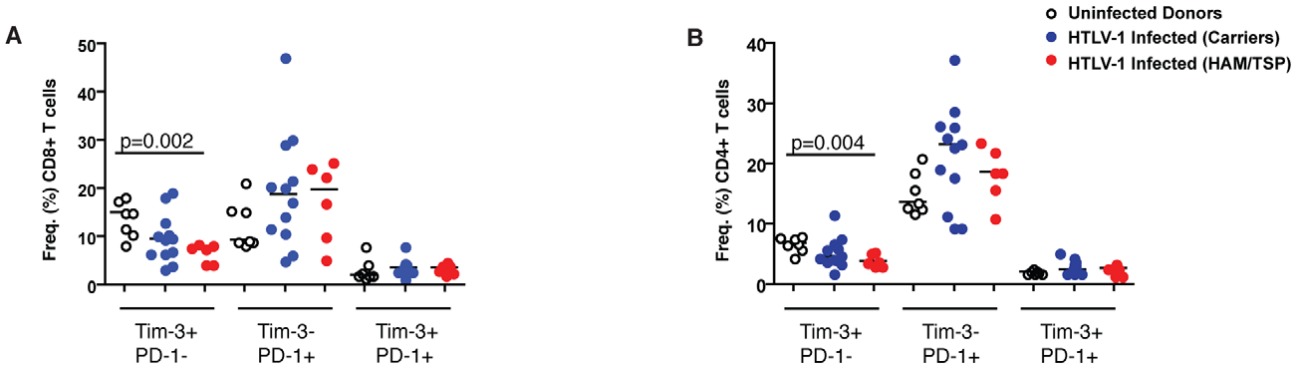
Tim-3 expression on T cells in HTLV-1 infection. Graphs show the frequencies of co-expression of Tim-3 and PD-1 on (A) CD8+ (left), and (B) CD4+ (right), T cells as assessed by multiparametric flow cytometry from PBMCs derived 18 HTLV-1 seropositive (12 asymptomatic and 6 with diagnosis of HAM/TSP) infected subjects and 7 HTLV-1 seronegative healthy uninfected donors from our initial recruitment. Statistically significant differences are reported as p<0.05.

Tim-3 and PD-1 are two cellular molecules expressed on T cells implicated in immune exhaustion. We evaluated the expression and co-expression of Tim-3 and PD-1 on T cells derived from HTLV-1 seropositive (both asymptomatic carriers and patients with the diagnosis of HAM/TSP) and seronegative controls to determine whether they were modulated in HTLV-1 infection. We observed a significant decrease in the frequency of Tim-3^+^ PD-1^−^ expressing CD8^+^ and CD4^+^ T cells among HTLV-1 seropositive subjects (CD8^+^: median 8.01%, IQR 5.42–10.50; CD4^+^: median 4.3%, IQR 3.50–5.99) compared to HTLV-1 seronegative controls (CD8^+^ median 15.10%, IQR 10.50–17.60; CD4^+^: median 6.84%, IQR 5.74–7.85) (Figure 1A and B). Patients with HAM/TSP (red circles) had significantly lower levels of Tim-3^+^ PD-1^−^ expressing CD8^+^ (p = 0.002) and CD4^+^ (p = 0.004) T cells compared to healthy uninfected controls (open circles). In contrast, the frequency of Tim-3^−^ PD1^+^ T cells trended to an increase in subjects with HTLV-1 infection (CD8^+^: median 18.80%, IQR 10.42–24.90; CD4^+^: median 20.70%, IQR 13.6–25.35) compared to healthy uninfected controls (CD8^+^: median 9.22%, IQR 8.97–15.50; CD4^+^: median 13.60%, IQR 12.7–18.6) (Figure 1A and B). Only a few T cells co-expressed both Tim-3 and PD-1, and no differences were observed between uninfected subjects and those with HTLV-1 asymptomatic infection or HAM/TSP patients. Using linear regression analysis we observed no association between the frequency of Tim-3 or PD-1 expression on CD8^+^ T cells in HTLV-1 infected subjects and proviral load. (p = 0.68; r = 0.1043; or p = 0.89; r = −0.03202, respectively).

### Distribution of Tim-3 Expression on HTLV-1-Specific T Cells

**Figure 2:**
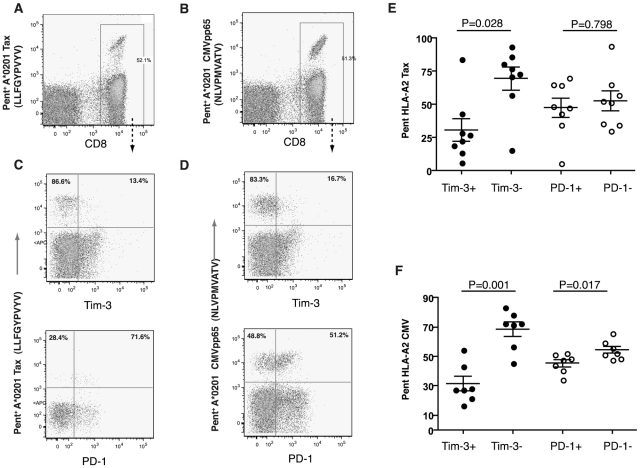
Tim-3 expression on HTLV-1-specific CD8+ T cells in HTLV-1 infection. PBMC from HLA-A*02+ chronically HTLV-1 infected individuals were stained with matched HLA pentamers presenting CMV and HTLV-1 epitopes, and with an anti-Tim-3 antibody. Shown are representative flow cytometry data from one HTLV-1-infected person using HLA-A*02 pentamers presenting the (A) HTLV-I-Tax 11–19 epitope and, (B) CMV-pp65 epitope ‘NLVPMVATV’. (C, D) Plots show co-expression of Tim-3 (upper panel) and PD-1 (lower panel) with the respective HLA-A*02 pentamers (Tax (left) and CMVpp65 (right)) from the gated CD8+ T population depicted in Fig 2 A, B. The percentages of cells in the upper left and right quadrants of the flow plots demonstrated in Figure 2C, D reflect only the percentage of pentamer expressing cells. The compiled expression data of the frequency of Tax (E) and CMVpp65 (F) pentamer cells on either Tim-3+ or Tim-3- and PD-1+ or PD-1- CD8+ T cells from 8 subjects are shown in Figure 2 E and F. Statistical analyses comparing pooled responses were performed using the Mann-Whitney test.

HLA- A*02 positive HTLV-1-infected patients have high amounts of circulating CD8^+^ T cells specific for an immunodominant HLA- A*02 -restricted epitope, HTLV-1 Tax 11–19 [20], [49], [50]. In HAM/TSP patients, these HTLV-1's Tax-specific CD8^+^ T cells correlate with HTLV-1 proviral load [23]. Among this cohort, we identified 15 HLA-A2 positive subjects (asymptomatic carriers, n = 9 and HAM/TSP, n = 6; Table 1), and evaluated the Tim-3 and PD-1 receptor expression on Tax-specific CD8^+^ T cells. Eight patients had Tax-specific CD8^+^ T cells (median 2.45%, IQR 1.11–5.31) as determined by specific pentamers. Among these patients we also observed HLA-A*02 -restricted CMVpp65 CD8+ T cells (median 2.49%, IQR 1.87–11.37). Interestingly, Tim-3 levels were dramatically reduced on CD8^+^ Tax 11–19-specific T cells (median 24.77%, IQR 15.2–39.54) compared to the expression of PD-1 (median 48.06%, IQR 36.81–65) (Figure 2A,C and E). We also evaluated Tim-3 expression on HLA- A*02 CMV specific T cells and found a similar pattern of expression with Tim-3 levels reduced on CD8^+^ CMV-specific T cells (median 27.62%, IQR 21.48–43.19) compared to PD-1 (median 47.70%, IQR 40.45–51.16) (Figure 2B,D and F).

### Relationship between the Functionality of Tax 11-19-Specific CD8^+^ T Cells and Tim-3 Levels

**Figure 3:**
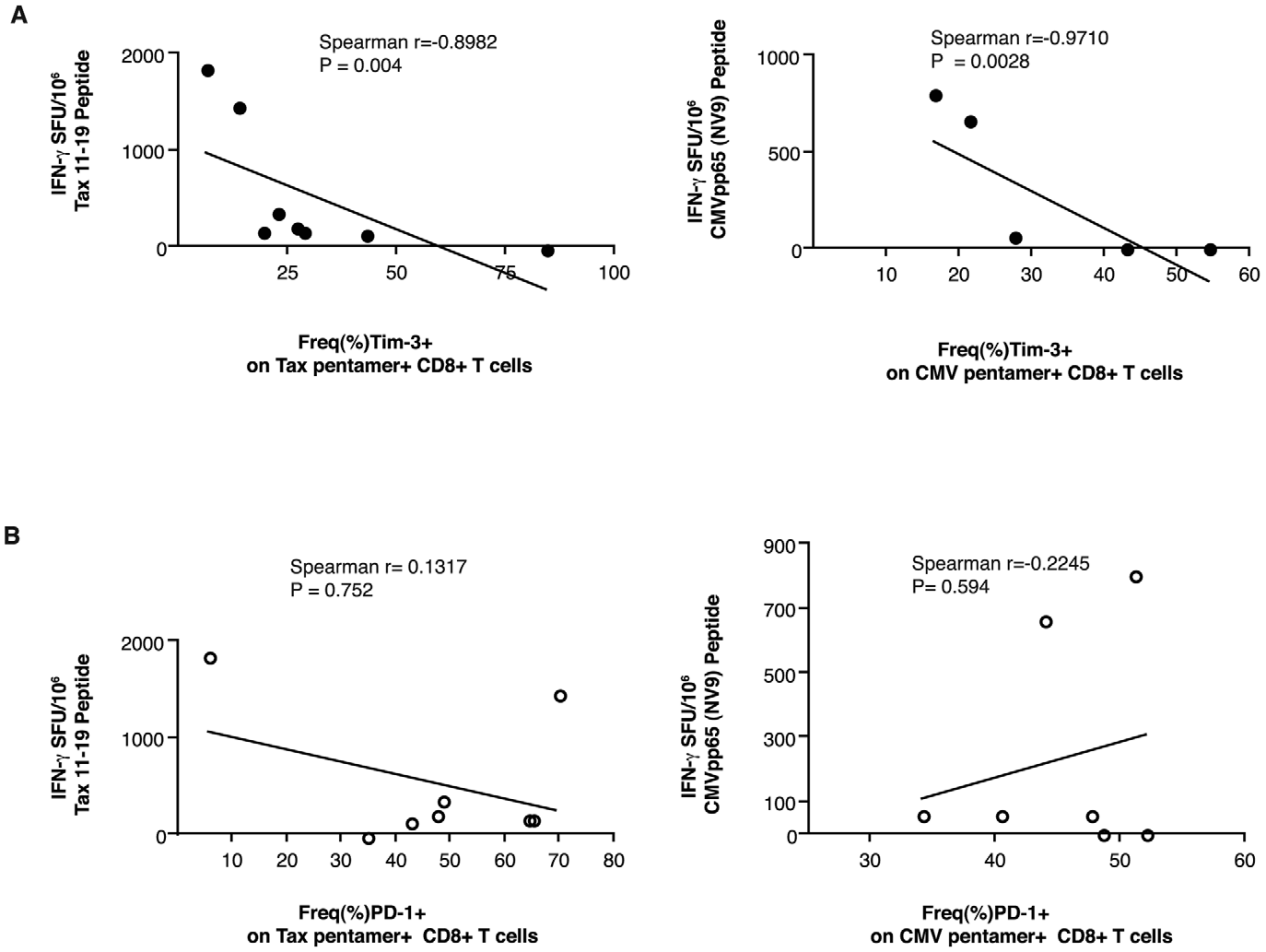
Association of Tax specific CD8+ T cells with effector responses. The graphs show the association between the frequency of Tim-3 (A) and PD-1 (B) expression on HLA-A*02 restricted Tax11-19 or CMV pp65 specific CD8+ T cells with the number of IFN-γ secreting cells (SFU/10^6^) in response to Tax 11–19 peptide or the CMV pp65 epitope. The Spearman rank test was used for correlation analyses.

To determine whether there was an association with Tim-3 or PD-1 levels on Tax 11–19-specific CD8^+^ T cells and their functionality, we evaluated the production of IFN-γ in response to the HLA-A*02-restricted Tax 11–19 immuno-dominant epitope and in comparison, the CMVpp65 epitope by an ELISPOT assay derived from PBMCs derived from 8 HLA- A*02 restricted infected individuals with Tax 11–19- and CMVpp65 specific CD8^+^ T cells (Figure 3). We saw no correlation between IFN-γ secretion and global PD-1 or Tim-3 expression on either the CD4^+^ or CD8^+^ T cells, irrespective of disease status (data not shown). The frequency of PD-1 expression on Tax-specific or CMV-specific CD8^+^ T cells also did not associate with the amount of IFN-γ secreted (r = 0.1317; P = 0.7520 and r = 0.2245; P = 0.594, respectively) (Figure 3B). However, we observed a statistically significant inverse correlation between the frequency of Tim-3 on both Tax-specific as well as CMV-specific CD8^+^ T cells and the amount of IFN-γ secreted (r = −0.8982; P = 0.0046; r = 0.9710; P = 0.0028; Figure 3A).

### Co-Expression of Tim-3 and Tax on T Cells in HTLV-1 Infected Cells

**Figure 4:**
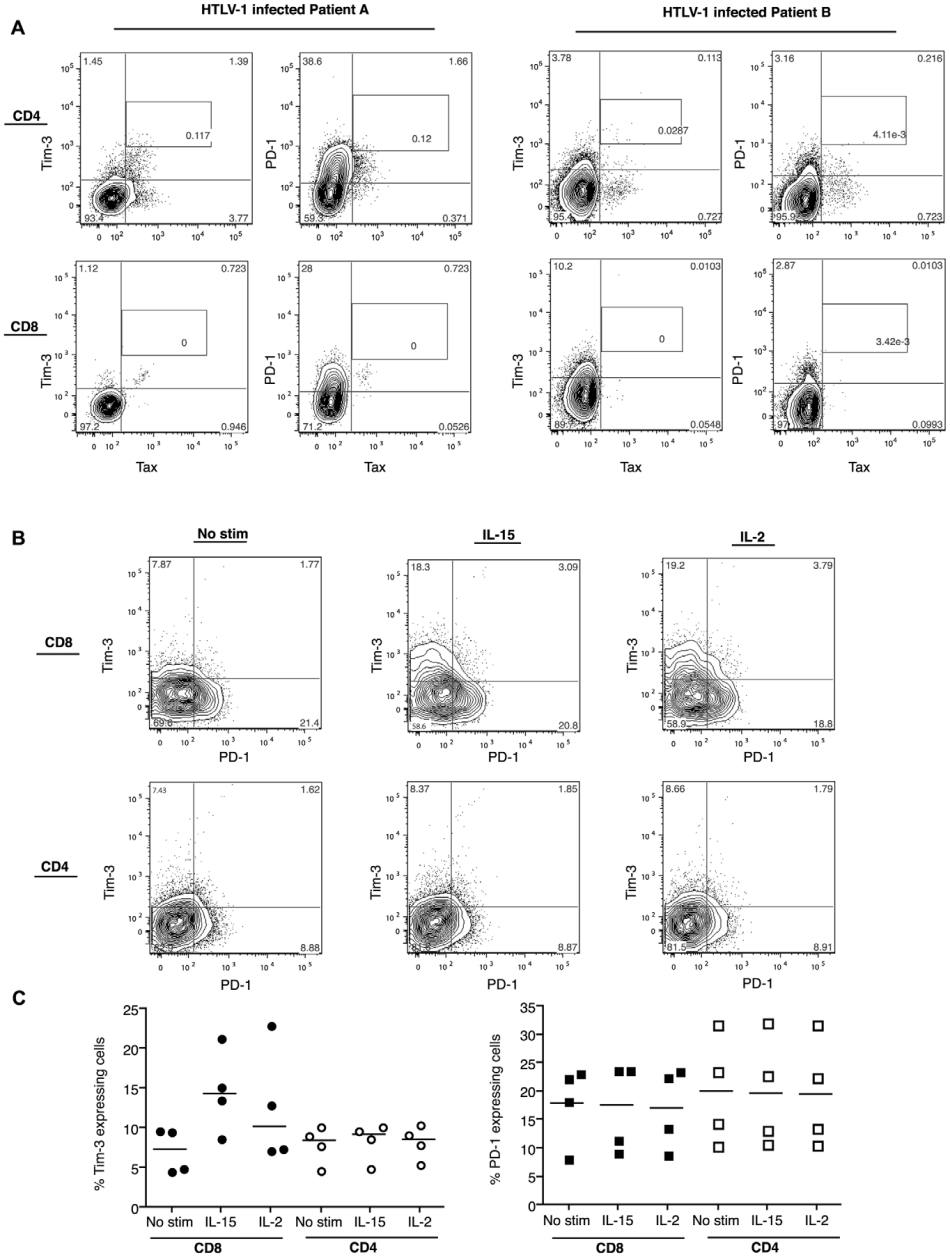
Tim-3, PD-1 and Tax co-expression on T cells. (A) Plots demonstrate representative co-staining for Tax, PD-1 and Tim-3 on CD8+ and CD4+ T cells by flow cytometry following 24 hours incubation for the induction of Tax in two representative HTLV-1 infected patients. An isotype control was used to delineate the measurements for Tax expression. (B, C) Plots and graph depict the co-expression of Tim-3 and PD-1 by the indicated cytokines after 12 hr in vitro culture of 1×10^6^ PBMC from 4 HTLV-1 infected patients. A representative donor is shown in B.

Tax expression marks HTLV-1 viral replication in both CD4^+^ and CD8^+^ infected T cells. We aimed to determine whether the downregulation of Tim-3 we had observed was occurring only among infected cells, or in bystander cells as well. We therefore co-stained for Tax and Tim-3 expression on T cells from HTLV-1 infected subjects. We also stained for PD-1 expression as a control. The culture of PBMC overnight did not alter Tim-3 or PD-1 expression levels on the HTLV-1-infected T cells (data not shown). We observed that Tax was expressed on PBMC from some subjects following 24 hours of culture and was detected on both Tim-3^+^ as well as Tim-3^−^ CD4^+^ T cells (Figure 4A). Similarly, Tax was present on both PD-1^+^ and PD-1^−^ T cells. We further identified a unique subset of Tax expressing CD4+ T cells that were Tim-3^hi^ and lacked PD-1 in most of the subjects expressing Tax (Fig. 4A). No difference in the pattern of co-expression between HTLV-1 seropositive asymptomatic patients and those diagnosed with HAM/TSP was observed.

### Elevated Tim-3 Expression by IL-2 and IL-15 Stimulated T Cells from HTLV-1 Infected Subjects

An increase in Tim-3 levels on T cells would potentially lead to a downregulation of T cell functionality. We therefore tested several gamma-chain associated cytokine mediators that could potentially modulate Tim-3 expression. We observed that IL-2, and especially IL-15, led to a prominent increase in the frequency of Tim-3 levels, specifically on the CD8+ T cell population after only 12 hours in culture (Figure 4B,C). No change in the levels of PD-1 expression were observed on both CD8^+^ and CD4^+^ T cells (Figure 4B,C).

## Discussion

CD8^+^ T cell dysfunction and/or exhaustion are common features of many chronic viral infections, including HIV-1 and HCV infections [29], [30], [31], [32], [33], [34], [35], [36]. The mechanisms of T cell dysfunction are complex, but are in part mediated by a distinct set of inhibitory receptors [27], [51]. A high, and sustained, expression of Tim-3 and PD-1, have emerged as hallmarks of T cell exhaustion in human viral infections, and blockade of these pathways can reinvigorate immune responses during persisting viral infections [29], [30], [33], [34], [36]. In this study, we report that CD8^+^ and CD4^+^ T cells in HTLV-1 infection express lower levels of Tim-3, and this was more pronounced in patients with HAM/TSP. Phenotypically, we observed that Tax HTLV-1-specific, HLA-A*02 -restricted CD8^+^ T cells consistently retain a lower frequency of Tim-3. We propose that this low expression of Tim-3 on HTLV-1 Tax-specific T cells may lead to a persistent and deleterious effector T cell pool leading to more inflammation.

The pattern of expression of PD-1 in HTLV-1 infection has recently been shown to be elevated on T cells in HTLV-1 carriers and also on CMV and EBV specific T cells in asymptomatic carriers compared to healthy controls [52]. This opposing relationship of PD-1 and Tim-3 expression on T cells in patients with HTLV-1 infection suggests that the downregulation of Tim-3 expression potentially leads to more vigorous T cell activity in the HTLV-1-infected individual, whereas PD-1 may not fully reflect T cell dysfunction, but rather an activated status of the T cell response to infection. Indeed the association between the frequency of Tim-3 and PD-1 levels with IFN-γ secretion in response to either Tax or CMVpp65 epitopes show remarkably different correlations. In a study by Petrovas and colleagues, it was apparent that PD-1 expressing T cells are able to secrete cytokines in response to viral peptides [39]. Our data suggests that PD-1 and Tim-3 on antigen specific CD8^+^ T cells are functionally different, and this may reflect a distinct stage of differentiation. PD-1 appears to mark early T-cell activation and exhaustion, while Tim-3 represents a more terminal stage of impairment.

The positive association between the frequency of HTLV-1's Tax-specific CD8^+^ T cells and HTLV-1's Tax mRNA load and proviral load is well documented [8], [53], [54]. Studies evaluating the phenotype of CD8^+^ T cells in HTLV-1 infection have been largely limited to characterizing the expression of T cell maturation and differentiation markers (CD28, CD45RO) [14]. Our data suggest that downregulation of Tim-3, rather than PD-1, marks global and Tax-specific CD8^+^ T cells, which are hyperfunctional. This contrasts with HIV-1 and HCV infections, where the expression of Tim-3 is increased, leading to a population of CD8^+^ T cells that are rendered dysfunctional both in terms of proliferative capacity and cytokine release as well as release of cytolytic granules [29], [36].

Surface receptors known to regulate T cell function like CD244 and PD-1 have been shown to be upregulated either directly due to Tax or indirectly due to the cytokine milieu [52], [55]. We postulate that either direct HTLV-1 viral components led to a downregulation of Tim-3, or as yet to be defined cytokine(s), suppress Tim-3 expression. In several human and murine studies, the manifestation of autoimmune diseases such as multiple sclerosis, have been attributed as a result of downregulated Tim-3 expression on T cells [56].

It still remains unclear how HTLV-1 infection sustains low levels of Tim-3 on T cells in infected patients and whether this is a cause or a consequence of disease progression. Multilayered mechanisms for this regulation may be occurring in the context of HTLV-1 infection. One strategy to reduce the T cells response would be through enhancement of the Tim-3 receptor for engagement with its cognate ligand. This could serve as a novel strategy to dampen the inflammatory inducing T cells. From our results, PD-1 engagement may not be as effective since both PD-1^−^ and PD-1^+^ cells retain the potential for CD8^+^ T cell lytic function.

A novel strategy to reverse or prevent the onset of neurological complications would be through dampening effector T cell functions. From our results, it appears the γ-chain cytokines elicited higher levels of Tim-3 on specifically on CD8^+^ T cells, and such a strategy could be harnessed to dampen T cell function in the HTLV-1 infected individual. Further work to understand the mechanisms for HTLV-1 disease progression and devise strategies to effectively prevent neurological complications will be needed. Targeted modulation of the Tim-3 pathway provides a viable model for this intervention.
